# The embodied mind extended: using words as social tools

**DOI:** 10.3389/fpsyg.2013.00214

**Published:** 2013-05-01

**Authors:** Anna M. Borghi, Claudia Scorolli, Daniele Caligiore, Gianluca Baldassarre, Luca Tummolini

**Affiliations:** ^1^EMbodied COgnition Lab, Department of Psychology, University of BolognaBologna, Italy; ^2^Institute of Cognitive Sciences and Technologies, National Research CouncilRome, Italy

**Keywords:** embodied cognition, extended cognition, tool-use, words as tools, language comprehension, social cognition, body schema, incorporation

## Abstract

The extended mind view and the embodied-grounded view of cognition and language are typically considered as rather independent perspectives. In this paper we propose a possible integration of the two views and support it proposing the idea of “Words As social Tools” (WAT). In this respect, we will propose that words, also due to their social and public character, can be conceived as quasi-external devices that extend our cognition. Moreover, words function like tools in that they enlarge the bodily space of action thus modifying our sense of body. To support our proposal, we review the relevant literature on tool-use and on words as tools and report recent evidence indicating that word use leads to an extension of space close to the body. In addition, we outline a model of the neural processes that may underpin bodily space extension via word use and may reflect possible effects on cognition of the use of words as external means. We also discuss how reconciling the two perspectives can help to overcome the limitations they encounter if considered independently.

## INTRODUCTION

The embodied-grounded (EG) view and the extended mind (EM) view of cognition and language are typically considered as rather independent perspectives. Aim of this paper is to show how the two views can be integrated considering the case of words in their relationship with the bodily space. Specifically, we will propose that words are a very peculiar kind of tool.

According to embodied views of cognition, cognitive processes are constrained by our body, that is, human-like cognition cannot occur independently of a human-like body. In the embodied view, cognition is not for knowing; rather, “cognition is for action” (Wilson, 2002). Proponents of grounded views make a similar argument but posit that the involvement of the body is not exhaustive of cognition, which is grounded in many ways (Barsalou, 2008). In fact, while initially the label “embodied” was used in a more comprehensive way, in the recent literature a slight distinction between embodied and grounded approaches, and between the terms “embodied” and “grounded,” is emerging (see Pezzulo et al., 2011; Fischer, 2012; Myachykov et al., in press). According to this view cognition can be grounded in multiple ways. These include not only bodily states, but also situations, actions, etc. (Barsalou, 2008; Pezzulo et al., 2011). In the following, we will use the term embodied and grounded cognition (EG) to refer to both approaches, since the distinction is not relevant for the proposal we will advance.

When it comes to language processing, EG views argue that language is grounded in perception and action systems (for reviews: Willems and Hagoort, 2007; Fischer and Zwaan, 2008; Gallese, 2008; Toni et al., 2008; Jirak et al., 2010; Borghi and Pecher, 2011, 2012; Glenberg and Gallese, 2012). Comprehending language would imply activating a simulation, consisting in a re-enactment of the previous interaction with objects, situations, etc., to which linguistic expressions refer.

In the last years another perspective on cognition, the EM view, is gaining credit, in particular in philosophy. The underlying idea, initially promoted by Clark and Chalmers (1998), is that the human mind is not wholly in our head/brain, but it is rather distributed in our brain, body, and external devices. These external devices (e.g., computers) have the power to complement and augment our internal cognitive processes (see Wilson, 2010).

In this paper, we will first discuss some general limitations of EG and EM views, then address some more specific limits of these views in understanding the role of language. We will then suggest that words can be understood as social tools, and explain why, in our opinion, this approach helps to reconcile EG and EM views of cognition and to overcome their limitations. Finally, we will discuss experimental evidence to support the Words as social Tools (WAT) proposal and we will outline a computational model to specify the neural mechanisms that might underlie the aforementioned processes.

## EMBODIED-GROUNDED AND EXTENDED VIEWS

Even though we favor an EG approach to cognition, we hold that EG theories have some problems (for critiques to aspects of the embodied approach, see Borghi and Cimatti, 2009, 2010; Chatterjee, 2010; van Elk et al., 2010; Wilson and Golonka, 2013). We will consider first some problems characterizing the EG approach in general, and then we will focus on the limitations of the EG approach to language, in particular to language comprehension. We will focus on content issues and not on methodological problems, as for example the problem of the lack of precise and unidirectional predictions, which in our opinion can be solved with a more extensive use of computational models (see for discussions on this problem Borghi et al., 2010; Chersi et al., 2010; Willems and Franken, 2012). Notice that our critiques might not necessarily concern all versions of EG views, which are sometimes rather different (see Goldman and de Vignemont, 2009, for an analysis of this). One major problem of EG views is the high risk of adopting the view that Clark (2008) has called “brainbound.” In this view, human cognition directly depends on neural activity, with the mind being modeled as inner and neurally realized. This position does not accept the idea that cognition might be distributed and extended beyond bodily borders. The brainbound view is not convincing for a simple reason, as explained by Noe (2009): “the subject of experience is not a bit of your body. You are not your brain. The brain, rather, is part of what you are” (pp. 7). In our opinion many versions of the EG view are too brainbound: they emphasize too much the role of the brain with respect to the body. This might seem paradoxical for an embodied approach: obviously no embodied view does fully neglect the importance of the body, but many EG approaches ascribe a too relevant role to the brain compared to the whole body, at the same time neglecting the possible role of body extensions. Similar critiques are expressed by Wilson and Golonka (2013) who claim: “The major problem with this research is that it again assumes all the hard work is done in the head, with perception and action merely tweaking the result.” (Wilson and Golonka, 2013, p. 11). van Elk et al. (2010) further deepen this point, arguing that in cognitive neuroscience embodied approaches are still cognitivist. We report their own words: “In cognitive neuroscience the notion that concepts are embodied primarily means that there is a correspondence between the brain activations associated with processing the referent of a concept and the processing of the concept itself. For instance, seeing a car and thinking or reading about a car involves the activation in comparable visual areas. Thus, the dispute between modal and amodal theories of language comprehension is basically a discussion about the representational vehicle of concepts (i.e., whether the representational vehicle of concepts is shared with neural resources used for perception and action). Both modal and amodal theories of language thus share a cognitivist notion of cognition in terms of discrete internal representations of the world” (van Elk et al., 2010, p. 3).

The second problem with many EG theories is that they do not sufficiently consider and emphasize the fact that the sense of body might be plastically rearranged. Body boundaries are treated as rather static while some studies have revealed that they are flexible and can be modified, for example through the use of tools, changing with our sense of body (see for example the special issue on the sense of body by Tessari et al., 2010). We will further address this problem in the rest of the paper.

When they deal with language, one major limit of EG views is that language is mainly conceived in its referential aspects. This way of conceiving language relies on the classical notion that knowing the meaning of a word is knowing what it refers to. Accordingly, the meaning of a word like “hammer” consists in the re-enactment of past multimodal experiences with the word referent, i.e. hammers. For example, according to the indexical theory (Glenberg and Robertson, 2000) words would index their referents in the world, which would be represented in terms of perceptual symbols (Barsalou, 1999). This referential view of language has a number of merits. First, it provided the instruments to contrast the propositional view, which was dominant in psychology and cognitive sciences (see Lakoff, 2012, for a description of the times before the idea of embodied cognition). In this view concepts and word meanings were seen as the product of a transduction process from sensorimotor to abstract knowledge. Knowledge would be represented in terms of amodal symbols only arbitrarily related to their referents, organized through syntactic combinatorial rules (e.g., Fodor, 1975; Pylyshyn, 1984). More recent non-embodied views posit that word meaning is a consequence of the statistical distribution of words in language (for an influential version, see Landauer and Dumais, 1997). However, today the necessity to contrast the statistical and the embodied view is not so critical, and conciliatory approaches have been proposed (see for example Andrews et al., in press).

Second, the influential research program based on these premises has inspired many studies, which have led to important and sophisticated experimental results (for reviews see Barsalou, 2008; Fischer and Zwaan, 2008; Gallese, 2008; Toni et al., 2008; Jirak et al., 2010; Borghi and Pecher, 2011, 2012). However, an embodied referential view is probably not sufficient to provide a thorough account of word meaning.

While in psychology and cognitive science the propositional view has dominated for a long time and the referential view was introduced by EG theorists as an alternative to it, in philosophy the referential view of language has been widely criticized since at least the seminal work of Wittgenstein (1953; see Noe, 2009 for a contemporary statement): the most widespread view in philosophy holds that, for example, we can speak about fawns even if we have never seen them since we can rely on the expertise of our community. Words are compositional and we can access the meaning of words of which we do not know or cannot see the referent thanks to the expertise of other members of our community. As Noe (2009) nicely argues, “meaning depends on the practice” (p. 90), and being able to use words corresponds to knowing what they mean. 

Curiously, while philosophical examinations have gravitated toward treating the practical nature of meaning, the referential view is still the predominant one in EG cognition theories. This has probably been due to the desire, on the part of EG proponents, to contrast the traditional propositional view, according to which words are arbitrarily linked to their referents. EG proponents have assumed that it was necessary to demonstrate that words are grounded, as their referents activate perception and motor systems.

Beyond the limit of the focus on referentiality, in our view the EG view of language has two further limitations given that it has neglected two other important aspects of words. The first concerns the social and public nature of words, the second the fact that words can be instruments for action. Words are social and public because, since they are a heritage of our speakers’ community, to be effective they require someone else’s presence, implicit or not. Indeed, speaking implies performing complementary actions in coordination with someone else (Clark, 1996). Words can be instruments for action since their use allows humans to modify the current state of the world, as it happens during tool-use. This point will be further developed in the course of the paper.

If EG approaches often tacitly assume a brainbound view of cognition, the most vigorous attack to this view derives from the idea that cognition is not limited to the boundaries of body/skull but is extended. In other words, “minds like ours emerge from this colorful flux as surprisingly seamless wholes: adaptively potent mashups extruded from a dizzying motley of heterogeneous elements and processes” (Clark, 2008, p. 219). According to the EM view, tools complement our mental abilities: for example, a diary complements our memory. As a consequence of this relationship between brain-body system and external tools, our mind would be distributed (Hutchins, 1995) across a variety of bodily parts and non-bodily devices (Clark, 2003; Thompson and Stapleton, 2009). One potential limitation of EM views, and possibly one of the reasons why they have encountered resistance, is their appeal to functionalism (Kiverstein and Clark, 2009) which might conflict with the assumptions of an embodied view of cognition (but see Clark, 2008, for a different position, which does not put the two approaches in contrast).

The EM approach holds a peculiar view of the relation between words and cognition. Words themselves are considered as external devices and as cognitive tools capable of augmenting our computational abilities (Clark, 1998). This view (e.g., Clark, 1998) has its roots in the seminal work of Vygotsky (1962) who underlined the role played by inner language and its scaffolding function supporting actions. However, in our opinion, one of the most interesting aspects of Vygotsky’s notion of inner language is that it involves the internalization of a phenomenon which is initially (and inherently) social and public and which augments our computational abilities. Such a social and public component is, however, underappreciated in the EM approach, which instead underlines the importance of language for developing thought and computational abilities.

Here we propose that EG and EM views can, and should, be integrated. Such integration will overcome their respective limitations when dealing with language: the limited focus of the EG view on the referential aspect of words and the neglect of the social dimension of words in the EM view.

## THE INCORPORATION OF PHYSICAL TOOLS

Even if it does not pertain to language, one line of research that may suggest how EG and EM views can be reconciled comes from recent work on the recoding of bodily space after tool-use. Below we will briefly review the behavioral, neural and computational literature on this topic and will then try to highlight why it is relevant for us.

Since Iriki’s seminal work with monkeys (e.g., Iriki et al., 1996), neuroscientific studies with humans have revealed that active tool-use can change the representation of space, in particular inducing an extension of the near space (Berlucchi and Aglioti, 1997; Berti and Frassinetti, 2000; Maravita and Iriki, 2004; Farnè et al., 2005; Osiurak et al., 2012).

The neural mechanisms underlying the extension of body representation caused by the use of a tool have not yet been identified (Magosso et al., 2010; Stout and Chaminade, 2012). Recently, some attempts mainly using computational modeling approaches have been proposed with the aim of identifying such mechanisms. Each proposed model sheds light on some important aspect underlying the phenomena. Ursino et al. (2007) and Magosso et al. (2010), for example, point out the involvement of visual-tactile cortical regions serving the representation of action affordances and action outcomes (including the parietal cortex, PC, and the pre-motor cortex, PMC) and Hebbian associative mechanisms to shape the body representation after using a tool. In particular, Ursino et al. (2007) claim that the enlargement of the peripersonal space after tool-use depends on an expansion of the visual receptive field of parietal bimodal neurons due to a strengthening of visual synapses through Hebbian mechanisms. In the same line the model proposed by Magosso et al. (2010) shows how different tool-use tasks lead to different re-sizing effects of the peri-hand space. The model also predicts that, after tool-use, a far visual stimulus acts as a near one, independently of whether the tool is present or absent in the subject’s hand. The authors validate this prediction by an *in-vivo* experiment. Other models focus on the role of sub-cortical areas (such as the cerebellum, see Arbib et al., 2009, and Imamizu and Kawato, 2012) in learning and storing internal models of body and environment after the use of a tool. Other ones suggest that memory processes are responsible for the dynamical aspects of tool-use during tool-body assimilation (Nabeshima et al., 2007; Nishide et al., 2009).

An open issue in the literature on bodily extension concerns whether the characteristic recoding of spatial perception also determines a change the body schema. We will briefly focus on this discussion since it is important for our view of language. One interesting distinction is between bodily extension determined by successful tool-use and incorporation following successful prosthesis-use. According to De Preester and Tsakiris (2009), tool-use does not determine changes in the sense of body-ownership, but only in motor and perceptual capacities (Botvinick, 2004). A crucial difference is the experience of completion: a non-corporeal object can be incorporated if it replaces something that originally was present, and now is missing. If the object cannot be assimilated to the pre-existing body-model (Tsakiris, 2010), true incorporation cannot occur. Beyond incorporation and use, there might be different degrees of relationship between ourselves and the objects. Some objects are perceived as external, while other objects provoke effects in our own sense of body. However, even objects perceived as completely external evoke motor responses (affordances), if they are close enough to our own body (Costantini et al., 2011b; Ambrosini et al., 2012; for a comprehensive review on affordances see Thill et al., 2013).

The same distinction between incorporation and use can also be applied to language. The question we will address in the following pages was initially proposed by Clark (2008, p. 39) in the following formulation: “Could anything like this notion of incorporation (rather than mere use)⋯ get a grip in the more ethereal domain of mind and cognition?” We will show how the notion of incorporation can be applied to the “ethereal” domain of language. Here words, and in particular their public and social dimensions, come into play.

## WORDS AS SOCIAL TOOLS: THE CASE OF SPACE

The idea that words can be conceived as tools is not completely new. Beyond Wittgenstein (1953), it has been proposed by a number of authors (Clark, 1998; Borghi and Cimatti, 2009, 2010, 2012; Mirolli and Parisi, 2009, 2011; Tylèn et al., 2010). However, different aspects of this idea have been stressed.

In *Philosophical Researches*, Wittgenstein (1953) highlighted the fact that words can have different and multiple functions, as tools in a toolbox. Clark (1998) spoke of the “magic” of words: words are external artefacts endowed with the power to augment and complement our computational abilities. According to him, while emphasis has been put on the communicative aspects of language, its computational role has been neglected, with the possible exception of Vygotsky who has underlined the role played by inner language and scaffolding to direct our actions.

The view we will present is slightly different. We agree that the computational role of inner language, intended as a guide for action, has not been considered enough. However, we intend to stress the role of other aspects of words that, despite the novel burst of interest for social neuroscience, have been neglected: the social and public role words possess. In order to be effective, words do not only need to refer correctly to objects or situations in the world. Language is also a powerful instrument for joint action. Words are tools, as they allow for the mental manipulation of information (Malt and Wolff, 2010). This in an individual and private use, as some authors have underlined. However, words have a peculiarity: to manipulate inner information we take advantage of a device that is social and public in its nature. For this reason we claim that words are “social tools.” Specifically, in this paper we will consider a special case of similarity between words as social tools and physical tools, concerning the relationship between space and body.

Words and physical tools share an important feature: both can be used to accomplish goals via external means, respectively, other people and objects, resulting in a change of the current state of the world (Glenberg and Gallese, 2012) and in an extension of our capabilities. Consider the case of words as tools that can be used to reach for something. We can reach objects with a physical tool (e.g., a rake), but also by asking somebody to bring them to us. Thus, in certain contexts the same goal can be reached either through tools, or through words. In some cases, words are even more powerful than tools. For example, they might allow us to reach very distant objects.

However, words work as tools only under the condition that other people collaborate. Even if our proposal is in debt with the pragmatics literature (e.g., Levinson, 1983) and with Austin (1962)’s idea that we do things with words, here we intend to make a distinction between advancing a request for an object and performing an action with a tool. These two activities share many similarities, but are also clearly different. An action with an instrument can be planned but fail, for example due to problems of the instrument, etc. Similarly, a request can be disattended, either because of problems in its formulation, or due to disruptions in communication, or scarce compliance on the side of the addressee. But people can decide to use tools to reach a goal on their own, without the presence of other individuals. This is not possible with words. The referent of a word can be found, but if other individuals do not provide a support, i.e., if the social dimension implied in word use is absent, the request will not succeed. Thus words are a peculiar kind of instrument: they work effectively only if other people are available and respond positively to our implicit or explicit request. What counts is the dynamic interaction they are able to promote (see Cooke et al., 2013, on team cognition). When performing activities which require coordination, such as lifting very heavy objects, we need to possess the sophisticated ability to understand others’ action plans, others’ willingness to collaborate, etc. (Marsh et al., 2009). Similarly, this ability should be present during language use as well, otherwise words, even if referentially correct, are not effective. In this respect, words constitute a bridge between ourselves, the environment and the others.

Here we propose that words and tools share a further similarity: we consider the possibility that when we use words to reach for something, word use expands the near space, modifying the representation of the relationship between our own body and the objects in space, similarly to what happens after tool use. The argument behind this hypothesis is the following: if words are similar to tools, then their use should lead to an extension of the bodily space, as it happens with real tools.

One could object that words and tools are substantially different, since tools are physical things in the world that we use with our bodies while words are not. We understand the objection, but the perspective we endorse is radically different: according to WAT (e.g., Borghi and Cimatti, 2009, 2012) not only tools but words as well can be considered as physical things. They are expressed through our bodies, be they spoken or written, and once pronounced or written they have a material and public existence, similarly to tools (Wittgenstein, 1953; Clark, 1998).

Now consider the relationship between words and body according to EG theories and the relationship between words and mind according to the EM view. EG theorists demonstrated that comprehending words activates the motor system. EM theorists propose that, as tools extend our body schema, “language extends our capacities for thought and therefore can be treated as extending our mind schema” (Noe, 2009). In fact, it has been shown that language modifies cognition, for example influencing perception and categorization (Wolff and Malt, 2010), in a flexible manner (Lupyan, 2012). But so far nobody has shown that word use might recode our bodily space with respect to objects, as it happens for physical tools. Notice that the parallel between words and tools is not only abstract and metaphorical; in contrast, we formulate the precise prediction, to be tested experimentally, that both words and physical tools have a specific effect on cognition, i.e., that their use determines an expansion of the bodily space representations. Demonstrating this would imply to apply the notion of incorporation to the “ethereal” domain of language. At the same time, it could help reconcile the EG and the EM view.

## WORDS AS SOCIAL TOOLS AND SPACE: EXPERIMENTAL EVIDENCE AND A MODEL

### EXPERIMENTAL EVIDENCE

Recent experimental evidence supports the idea that words can be considered as tools that extend the bodily space.

Scorolli et al. (2011; submitted) and Scorolli and Borghi (2012) demonstrated with a kinematics study that word use modifies spatial perception. Participants, children and adults, observed objects located in the peripersonal, extrapersonal or far and “border” space. For operational reasons we defined “peripersonal,” or “near,” as the space reachable extending the arm (but see the discussion on the problems of this definition due to the plasticity of the near space made by Longo and Lourenco, 2006; Lourenco and Longo, 2009), “extrapersonal,” or “far,” as the non-reachable space, and “border” as the space reachable extending the arm and the back. Before and after training, subjects were asked to produce explicit verbal estimations on objects’ distances, or to throw a toy-car toward objects’ locations. During the training phase participants had to reach and grasp the “right” object and to put it in a box provided by different shaped holes. If the right object was too far, they could use a tool (a rake), press a button or use a linguistic label, pronouncing the object noun; all instruments were effective in reaching the goal. We introduced the button since we were interested in comparing the rake and the button, i.e., two instruments that, differently from words, do not imply a social context to be used. While participants hold the rake in their hands, the button has an arbitrary relation to the object, similarly to a word: once pressed, the object appears. In the last years, few studies have shown that even arbitrary relationships with a target can modify the perception of peripersonal space. Davoli et al. (2012) have shown that remote interactions with a target, for example illuminating the target object with a laser pointer, caused an extension of the perceived space. In the same vein, Bassolino et al. (2010) demonstrated that frequent use of a computer mouse determined a spatial extension. The difference between a button, i.e., a device that is arbitrary linked to the object to be reached, and a word is that the last one implies a social dimension.

The results of the study revealed that after training, even if the verbal estimations changed slightly, the car was thrown significantly closer than before the training. This indicated an extension of the reachable space, not modulated by the instrument kind.

As other studies on extended body, this work suggests that the distinction between near and far space is plastic and flexible. However, here the extension was brought about not only by physical tools but by immaterial ones as well, i.e. by words. The social dimension implicit in words made this possible: pronouncing an object name implies evoking somebody else performing a complementary action, helping us reach a distant object. Thus words, like tools, help us act in the world and influence our way of representing bodily space with respect to objects (Gianelli et al., 2013). However, with words, our operational space becomes larger because of the presence of others. Even if we propose that the social dimension is intrinsic in word use *per se*, we predict that the results will be stronger, i.e. the spatial extension with words will be more marked, in presence of another person. In particular, this extension should be particularly marked if the other person is close to the object, is looking directly at the participant and demonstrates through gestures and posture to be open to the interaction (see Innocenti et al., 2012; Scorolli et al., 2012). We predict, instead, that if the other person is not close to the object, and the body posture and the facial expression of the other are not expressing compliance, the effects of words will be reduced, given that the request is less likely to be attended. In sum, Scorolli et al. (2011; submitted) have shown that words alone are effective in modifying the bodily space. However, we predict that their effect will be more marked in a context in which the social dimension is emphasized, thanks to the real presence of another person.

These results are complementary with those obtained by Costantini et al. (2011b). Previous evidence demonstrated that objects afford actions only when presented in the peripersonal space e.g., Costantini et al. (2011a). The novelty of the study by Costantini et al. (2011b) consisted in showing that when the object was outside subject’s reaching space but within an avatar’s reaching space, it evoked affordances as well. According to the authors, this indicates that an interpersonal body representation is formed in which one’s own arm reaching space is mapped with that of others’. Notice that an avatar might evoke the presence of another person, but the effects it produces might not be as strong as those elicited by the presence of a real other.

However, these findings together with those by Scorolli et al. (2011; submitted) and Scorolli and Borghi (2012), in which words refer only implicitly to the presence of another person, suggest that the subject’s representation of reaching space is actually extended. Importantly, in the study by Scorolli et al. (2011; submitted) the other person plays a complementary role as he/she is implicitly evoked to perform an action one cannot perform alone (Newman-Norlund et al., 2007).

In sum: it has been suggested that active tool-use determines a progressive incorporation of the tool within the body schema (Iriki et al., 1996; Povinelli et al., 2010). The analogous extension of the operational space found after the rake, the button and the word use suggests that the reaching space extension is not due to the possibility of the tool to be integrated into the body schema, but to the goal-directed character of the action (Hommel et al., 2001; Massen and Prinz, 2009). However, some issues remain open.

The studies discussed so far indicate that words, similarly to real tools, determine a plastic modification of the reaching space, even if they cannot be integrated into the body schema as tools do. However, the evidence we reported concerns concrete words, and specifically words with specific referents endowed with a precise spatial location. One could ask whether the claim that words are tools can be generalized, i.e., whether other kinds of words can determine variations in the bodily space. Even if we are not aware of any evidence, we can speculate that even words like “the” or like “freedom,” which do not have a specific concrete counterpart, can expand our near space (for work on mapping between demonstratives such as “this” and “that” and near and far space, see Coventry et al., 2008, 2012; Bonfiglioli et al., 2009). As we say something to somebody else through words we somehow create a novel, shared space. This should happen with each word, as each word is pronounced to be heard by somebody else. However, while we reported evidence showing that concrete words expand the peripersonal space, the possibility that this is true for other kinds of words is currently a speculation, and further research is needed in order to demonstrate it.

A further question one could raise is the following: do intransitive gestures as well induce an extension of the near space, similarly to tools? Indeed, for communicative gestures to succeed, we need that others are available and ready to collaborate, as it happens with words. Compared to gestures, however, words have a number of advantages: (a) they are typically more specific than gestures (e.g., I can point to an object I would like to receive, but the context might not help you to identify the precise object: this potential problem can be easily solved using the appropriate word); (b) they are arbitrarily related to their referents, and this allows more freedom of action; (c) also thanks to b, they are less tightly anchored to a specific context and situation. Normally gestures coexist with words, even if they can have a separate meaning (McNeill, 2000; Kendon, 2004). Furthermore, it has been shown that gestures do not develop imitating others, but emerge in an autonomous way and are integrated in speech, probably because they facilitate thinking (Bates, 1976; Capirci et al., 1996; Iverson and Goldin-Meadow, 1998). On this basis we can advance the prediction that combinations of gestures and words would increase the effect with respect to words alone. As to the sign language, where gestures directly substitute words, we predict a similar effect as the one obtained with words. But consider the case in which gestures are not coupled with words but used as substitutes for them. In this case our predictions are not so straightforward, and further research is needed to investigate this important issue (for relevant work, see De Stefani et al., under review).

### TOWARD A COMPUTATIONAL MODEL OF WORDS AS SOCIAL TOOLS

Although the models reviewed in the Section “The incorporation of physical tools” give important insights on the brain mechanisms underlying the adaptation of body representation after using a tool, they do not deal with the question of the possible neural mechanisms underlying the processes of words as tools. To address this problem, it is crucial to consider three key aspects not yet considered by previous models: (a) the brain has a hierarchically (soft)modular organization (Meunier et al., 2010; Houk, 2011; b) such organization pivots on anticipatory/goal-based representations of actions at multiple levels (Hamilton and Grafton, 2008; c) words are grounded on the same (or contiguous) neural representations sub-serving action (for reviews, see Martin, 2007; Jirak et al., 2010).

**FIGURE 1:**
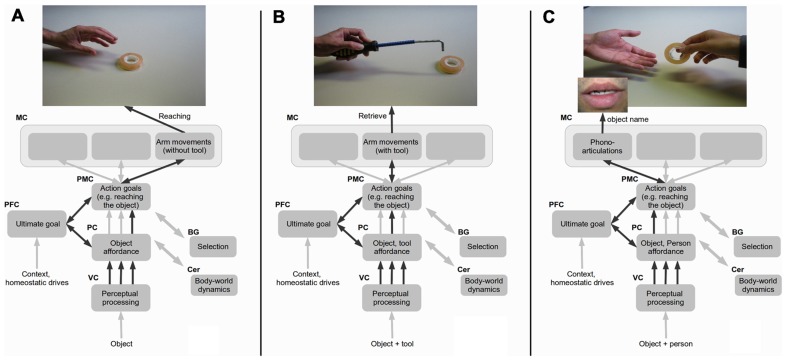
**A model of tool-body assimilation**. In the model, the visual cortex (VC) performs basic visual processing; the parietal cortex (PC) extracts affordances based on body/world relations and encodes abstract action goals; the pre-motor cortex (PMC) prepares actions based on more specific action goals; the pre-frontal cortex (PFC) encodes the agent’s ultimate goals based on context and internal homeostatic regulations, and on this basis contributes to form action goals in the PMC and the PC; cortico-cerebellar (Cer) loops simulate body-world, dynamics; cortico-basal ganglia (BG) loops underlie action and affordance selection processes. **(A–C)** represent a possible progression of development of representations from body actions, to tool-mediated actions and words-as-tool actions, all relying on the same macro brain areas and on partially overlapping local neural modules (cell assemblies). Multiple PC-PMC arrows represent multiple affordance-action options and the black arrow the most active within these.

A bio-inspired neural architecture based on these points is sketched in **Figure 1**. The overall model architecture is built on the model of Caligiore et al. (2010), capturing important aspects of hierarchical brain organization. Even if the model proposed here is not computationally implemented, the discussion of its design features allows us to unveil important aspects overlooked by current models on tool-body assimilation. These aspects could be important to investigate the neural mechanisms underlying the notion of words as social tools.

**Figure 1A** represents the fact that the vision of an object in the peripersonal space evokes several potential affordances (encoded by the neurons of PC) and actions (encoded by the neurons of PMC) selected based on BG and local competitions (Cisek, 2007). Importantly, some neurons of these areas represent affordances and actions in terms of expected outcomes (Hamilton and Grafton, 2008) or goals (“distal goals,” Umiltà et al., 2008), such as “reaching the object,” rather than in terms of detailed movement commands encoded in the motor cortex (MC). The pre-frontal cortex (PFC), which encodes the agent’s ultimate goals based on the internal and external context, exerts a top-down biasing effect on the formation of proximal goals and on the selection of different affordances and actions taking place in the PC and PMC and ultimately leading to perform specific movements (MC).

The mechanisms of affordance and action selection based on goals are crucial to explain the modulation of neural representations when a tool is used to reach far objects. The key idea is that the neurons of PC/PMC encoding affordances and actions in *terms of expected effects can allow the abstraction of the specific aspects of actions pertaining to the use of the limb or the tool*. For example, **Figure 1B** shows that, when using a tool to reach the object, PC neurons might encode the salient features of both the target and the tool while PMC neurons might encode the “reach the target” goal: as these representations have many features in common (same object and context, similar effect, similar attentional focus on the object, etc.) with those activated when reaching without a tool, the neural populations encoding them might strongly overlap and form Hebbian associations. These might lead to change the representations related to space.

The effects of words as tool on space representation might be due to these mechanisms and to the fact that words are grounded in the same neural structures underlying perception and action (Caligiore et al., 2010). **Figure 1C** shows this with an example where the object is in the extrapersonal space but another person is close to it. In this case, the use of a phono-articulation of a word (e.g., the name of the target directed at a caregiver in childhood) might produce the same outcome of a direct reach. This and the similarities of context, intentions, target, or even (failed) reaching movement, might cause an overlap and association between the space-related representations active in the two conditions. The fact that heard words may further compact sensorimotor representations (Mirolli and Parisi, 2009) would strengthen this process. This might warp all representations of space incorporating “reachability” information and lead to effects such as those observed in our study. 

Possible alternatives to our view could refer to the fact that the neural basis for language comprehension and tool-use might to some extent differ. As it is well known, the ventral stream plays a major role for semantics and language processing, whereas the dorsal stream is crucial for action preparation and execution (e.g., Chao and Martin, 2000; Johnson-Frey et al., 2005), processes very important for tool-use. There is also clear evidence of dissociations between language and praxis in neuropsychological patients (e.g., Buxbaum et al., 2001; Humphreys and Forde, 2001).

We do not think that our proposal is really weakened by these arguments, for at least two reasons. First, recent literature has smoothened the distinction between ventral and dorsal streams (see for example Goodale and Westwood, 2004). Some authors have shown the many interactions between the two routes (Gallese et al., 1999). Furthermore, a sub-distinction between a dorso-dorsal and a dorso-ventral route has been proposed (e.g., Rizzolatti and Matelli, 2003). Accordingly, words referring to action would be processed in the dorso-ventral rather than in the ventral stream (see proposals by Binkofski and Buxbaum, in press; Borghi and Riggio, 2009; Marino et al., 2013).

More generally, our aim is to show that in some conditions words can change some of our internal brain representations as is done by tools (for an analysis of shared brain mechanisms between complex tool-use and language, see Frey, 2008), but not that the caused changes are identical in the two conditions.

At a more basic level, here we do not intend to argue that language use equals tool-use in all respects. In line with theories of reuse (e.g., Anderson, 2010) we think that language is grounded in the sensorimotor system, but that, being at a higher abstraction level, modifications and constraints are introduced (for developing this argument, see Borghi, 2012). In synthesis, our aim is to show that words are tools, but they are not only tools.

## CONCLUSION

Words are first encountered as objects. They are peculiar objects, though, because they implicitly refer to a social and public dimension and because they are immaterial ones. Later they become internalized (Vygotsky, 1962). The capability to use (inner) language modifies our internal processes; language is a powerful means to reconfigure our mental abilities and capability of control. Therefore words help us in “self-engineering” ourselves, to perform better in our ecological niche. But when we produce them, words are also objects outside from us. Differently from the physical tools that, when used, recode the spatial relationship between our body and the world, words are part of the ethereal world of cognition. Even if they are immaterial, we have suggested that words are both extended and embodied. They are both extended and embodied because their use determines a remapping of the relationships between our body, the objects and the space.

The evidence that EG theorists have collected shows that words are embodied and grounded in our sensorimotor system. However, so far EG research has been exceedingly focused on words’ referents and on how their meaning is represented in the brain, while neglecting what can be achieved through words. Seeing words as tools that extend our near space allow us to overcome these limitations.

At the same time, EM theorists have shown that words can be used as tools that augment our computational potentialities, and that meaning is not limited to what is represented in the brain. However, the EM perspective has insufficiently explored the social and public role words play. As we have shown, the remapping of the bodily space we found with words is granted by the fact that words imply the presence of others: somehow our own space becomes larger as it incorporates the space of others. These implied others complement our abilities, and we call them into play by means of words.

In sum, we think that the idea that words work as social tools that extend our near space can help combining two very promising and sophisticated perspectives, the EG and the EM views.

We agree with Clark (2008) when he invites us “to cease to unreflectively privilege the inner, the biological, the neural.”(p. 218). Accepting this invitation does not imply avoiding to ascribe value to the inner, the biological, the neural. In contrast, it permits the combination of an EG and an extended perspective on cognition in which the mind emerges “at the productive interface of brain, body, and social and material world.” Treating words as social tools highlights exactly this.

## Conflict of Interest Statement

The authors declare that the research was conducted in the absence of any commercial or financial relationships that could be construed as a potential conflict of interest.
